# Does Multimorbidity Influence the Occurrence Rates of Chronic Conditions? A Claims Data Based Comparison of Expected and Observed Prevalence Rates

**DOI:** 10.1371/journal.pone.0045390

**Published:** 2012-09-17

**Authors:** Ingmar Schäfer

**Affiliations:** Department of Primary Medical Care, University Medical Center Hamburg-Eppendorf, Hamburg, Germany; University of Ottawa, Canada

## Abstract

**Objective:**

Multimorbidity is a complex phenomenon with an almost endless number of possible disease combinations with unclear implications. One important aspect in analyzing the clustering of diseases is to distinguish between random coexistence and statistical dependency. We developed a model to account for random coexistence based on stochastic distribution. We analyzed if the number of diseases of the patients influences the occurrence rates of chronic conditions.

**Methods:**

We analyzed claims data of 121,389 persons aged 65+ using a list of 46 chronic conditions. Expected prevalences were simulated by drawing without replacement from all observed diseases using observed overall prevalences as initial probability weights. To determine if a disease occurs more or less frequently than expected by chance we calculated observed-minus-expected deltas for each disease. We defined clinical relevance as |delta| ≥ 5.0%. 18 conditions were excluded because of a prevalence < 5.0%.

**Results:**

We found that (1) two chronic conditions (e.g. hypertension) were more frequent than expected in patients with a low number of comorbidities; (2) four conditions (e.g. renal insufficiency) were more frequent in patients with many comorbidities; (3) six conditions (e.g. cancer) were less frequent with many comorbidities; and (4) 16 conditions had an average course of prevalences.

**Conclusion:**

A growing extent of multimorbidity goes along with a rapid growth of prevalences. This is for the largest part merely a stochastic effect. If we account for this effect we find that only few diseases deviate from the expected prevalence curves. Causes for these deviations are discussed. Our approach also has methodological implications: Naive analyses of multimorbidity might easily be affected by bias, because the prevalence of all chronic conditions necessarily increases with a growing extent of multimorbidity. We should therefore always examine and discuss the stochastic interrelations between the chronic conditions we analyze.

## Background

Research on multimorbidity is often guided by the assumption that multimorbidity is more than just the sum of single diseases [1]. But what then exactly is multimorbidity? In most studies multimorbidity means the presence of several chronic diseases in one person for a longer period of time [2]. It is highly prevalent in the elderly population and may result in decline of functional status, lower quality of life, higher mortality, increased health care utilization and therefore rising costs of care [3]. It is presumed that multimorbidity causes a different dimension of suffering. On the one hand the combination of diseases might lead to a higher illness burden than the single diseases; on the other hand chronic conditions might share symptoms and/or risk factors [1].

We should note that multimorbidity is a complex phenomenon with an almost endless number of possible disease combinations with unclear implications. Recent research has described and grouped these combinations by introducing triads of chronic conditions [4], or multimorbidity patterns resulting from cluster analysis [5] or factor analysis [6]. Despite these efforts the process and pathway of multimorbidity is still not known [7].

Multimorbidity may occur in case of random coexistence of diseases, merely statistically significant associations or causal interrelations between chronic conditions [8]. One important aspect in analyzing the clustering of diseases in individual patients is to distinguish between random coexistence and statistical dependency.

We presume that in a large number of cases clustering of chronic conditions is determined by chance alone. It is non-trivial to discriminate statistically between random co-occurrence and statistical association. For this reason we developed a model to account for random coexistence based on the stochastic distribution in our sample.

The aim of this study is to determine if multimorbidity influences the occurrence rates of chronic conditions. For this reason we analyzed if there is an association between the number of diseases of a chronically ill person and the risk of gaining selected chronic conditions. We can define that multimorbidity has no effect on this process if the gain of new diseases is determined by chance alone, e.g. a healthy person would have the same chance of gaining diabetes mellitus type 2 as a person who already has 8 diseases.

### The statistical problem

Statistical independence between chronic conditions can be described as an urn model. Each of the 46 diseases in our analyses is represented by a ball in an urn. The process of gaining diseases then corresponds to randomly drawing balls from this urn. For each additional disease a new ball is drawn. As the diseases all have a different prevalence each of the balls would also have a different probability of being drawn (e.g. because of a different weight). The balls are not returned back into the urn once they are drawn. For this reason the probability for drawing each ball changes in the next draw depending on the probability of the balls that were drawn before. If we want to know the probability of each of the diseases in the first draw, the second draw, the third draw etc. we have to sum up all the single probabilities.

An example: We can imagine a population with one to three diseases from a list of four. Chronic condition no. 1 (CC1) has a prevalence of 60%, CC2 25%, CC3 10% and CC4 has a prevalence of 5% among the patients with only one disease. As the chronic conditions are supposed to be statistically independent from each other the probability for each draw (p) is the same as the prevalence in the first draw (P_1_). For CC3 this is expressed by the following formula: 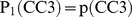
(1)


If we take a look at the prevalence in persons with two diseases (P_2_) there are the following possibilities: 1) A person could gain CC3 in the first draw or 2) he or she could first gain CC1 or 3) CC2 or 4) CC4 and then CC3 in the second draw: 
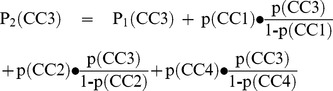
(2)


If want to determine the prevalence in persons with three diseases (P_3_) it gets a little more complicated. A person could gain CC3 as first or second disease, or 5) first get CC1 then CC2 or 6) first CC1 then CC4 or 7) first CC2 then CC1 or 8) first CC2 then CC4 or 9) first CC4 then CC1 or 10) first CC4 then CC2 and then CC3 in the third draw: 
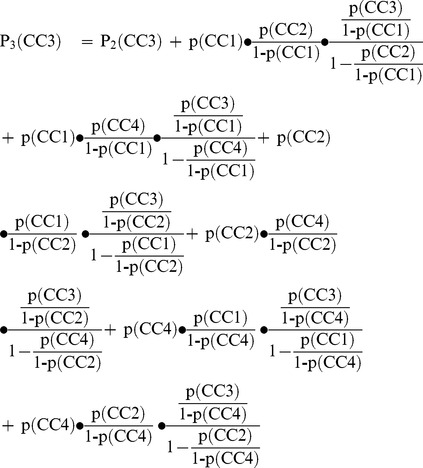
(3)


**Figure 1:**
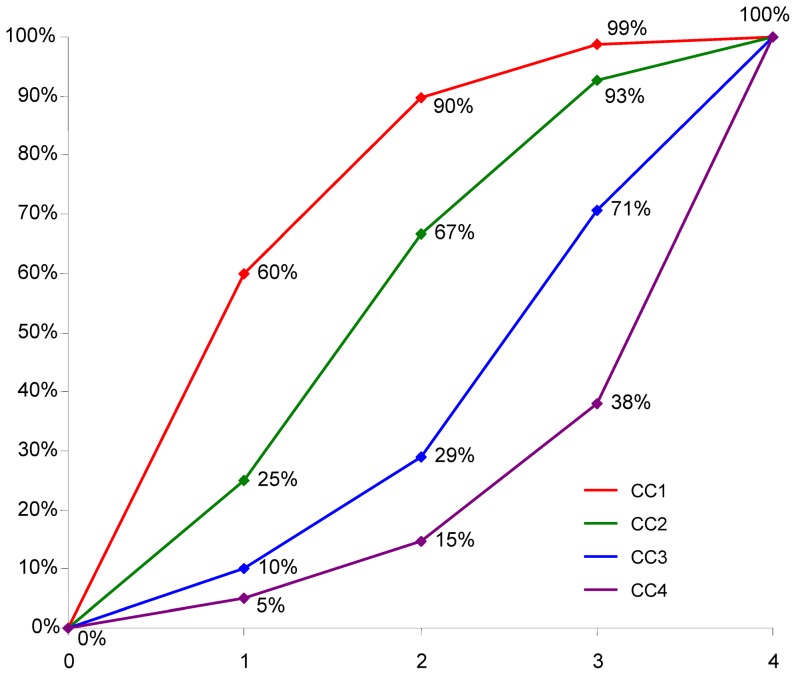
Prevalences by total number of diseases for four (thought up) chronic conditions (CC1 to CC4).

The probability of having one of the four diseases in each draw is shown in Figure 1. For CC3 the prevalence increases from 10% to 29% to 71% if we have complete statistical independence between prevalence and the total number of chronic conditions one person has. As a matter of course the prevalence of each disease would be 0% in a healthy subsample and 100% in a subsample of persons with four diseases from our list of four.

The association of a chronic condition with multimorbidity can be expressed by relative risks for multimorbidity in which the prevalence in the non-multimorbid population is compared to the prevalence in the multimorbid population [4]. If use our above example and define that 1,000 persons have one disease, 400 have two and 150 persons have three diseases, we can calculate these risk ratios for each disease. In our example we would define multimorbidity as having at least two chronic conditions, so that the non-multimorbid sample consists of all patients having exactly one chronic condition. The risk ratios for multimorbidity are shown in Table 1. Although all four diseases are statistically independent from multimorbidity they have different risk ratios ranging from 1.5 to 4.2. If we would also have healthy persons and/or persons with all four diseases in our sample, the risk ratios would even be much higher.

**Table 1:** Risk ratios for multimorbidity in four (thought up) chronic conditions (CC1 to CC4).

	1 disease (n = 1,000)	2 diseases (n = 400)	3 diseases (n = 150)	Multimorbid (2 or 3 diseases)	risk ratio for multimorbidity
CC1	60.0%	89.8%	98.8%	92.3%	1.5
CC2	25.0%	66.6%	92.6%	73.7%	2.9
CC3	10.0%	28.9%	70.7%	40.3%	4.0
CC4	5.0%	14.7%	38.0%	21.1%	4.2

## Methods

The analyses are based on the comparison of two data sets. The first data set (“observed data”) consists of ambulatory data of the Gmünder ErsatzKasse, a German statutory health insurance company with 1.7 million insurants (in 2008), which corresponds to 2.4% of the statutory insured population [9]. The dataset contains pseudonymous data from every insured member of this company. We used a sample of all persons aged 65 years and older who were permanently insured during the year 2006 and had at least one and not more than 12 chronic conditions. Healthy patients have been excluded because by definition all diseases have a prevalence of 0% in healthy subjects and therefore they did not contribute any information to the analyses. Patients with more than 12 diseases have been excluded because of a low sample size in these subsamples (< 1,000 patients) which might lead to biased results in diseases that have a rather low prevalence.

The second data set (“expected data”) was generated in order to model the chronic conditions as statistically independent from multimorbidity. As we used 46 chronic conditions and 12 draws we would have had to calculate hundreds of extremely complex equations that each included many thousands of possibilities. As each of these equations also would be unique and had to be programmed separately it was not possible to calculate the expected prevalences by this brute force method. Instead, a data set of 500,000 hypothetical persons was generated by simulation, which was based on our stochastic model. We used the prevalence data found in the observed data set as probability weights for the diseases. In our simulation we repeatedly produced sequences of diseases by drawing without replacement from all observed diseases. Each simulated patient gains a total of 12 diseases. The order of gaining the diseases is stored. For this reason the expected data set contains information about patients with one through twelve diseases. As we presume that chronic conditions can occur “earlier” or “later than expected” in the course of multimorbidity we used the observed overall prevalences, i.e. the mean prevalences in all persons aged 65+ in our data set (instead of the mean prevalences in persons aged 65+ that have only one disease) as initial probability weights.

The analysis of morbidity was based on a list of 46 defined diagnosis groups of chronic conditions based on ICD-10 codes. The methods for compiling this list have been described elsewhere in detail [4]. In short, we used the most frequent conditions reported in GP surgeries [10], assessed them for chronicity using a recent expert report [11] and amended this list for all chronic conditions with a prevalence ≥ 1% in the age group ≥ 65 years in the data set of the Gmünder ErsatzKasse in 2006. ICD-10 codes were grouped together if diseases and syndromes had a close pathophysiological similarity and if ICD codes of related disorders were used ambiguously by coding physicians in clinical reality, respectively. Prevalence, gender-specific rank order and ICD-10 codes of the diagnosis groups have been published in another paper [6].

All problems under management by physicians within the statutory ambulatory care have to be coded in ICD-10 and forwarded to the health insurance companies as regulated by German law in §295(1) SGB V and §44(3) of the Federal Collective Agreement within the statutory health insurance system in Germany [12]. Each problem must be represented by one or more ICD-10 codes. We only included diagnoses that were covered by the list of 46 diagnosis groups and had been coded in at least three out of four quarters (three month periods) within the year 2006. This criterion was chosen in order to increase the validity of the diagnoses based on claims data by avoiding transitory or even accidental diagnoses.

Multimorbidity is often used as a dichotomous variable, i.e. the sample is divided around a cut-off point of 2 or 3 diseases. This definition results in a loss of information that can be avoided. For this reason we used multimorbidity as an ordinal variable by dividing the observed and the expected data set into 12 subsamples. Subsample 1 consists of all persons who have exactly one chronic condition. All persons with exactly two chronic conditions are in subsample 2 etc.

To determine if a disease occurs more (or less) frequently than expected by chance we compared our two data sets by calculating “observed minus expected deltas” for each disease in each subsample, e.g. we compared the observed prevalence of hypertension in patients with two diseases with the expected prevalence of hypertension in this patient group. We report the overall prevalences (over the subsamples 1 to 12), observed and expected data for the extremes (subsample 1 and subsample 12), the highest deltas for each disease and the subsample in which the delta is highest.

Because of the large sample size we did not test for statistical significance, but instead used a criterion for clinical relevance. We defined that a disease is associated in a clinically relevant extent with the total number of chronic conditions if the absolute value of the observed minus expected delta in one subsample is 5.0% or more. For all diseases with a clinically relevant delta we compared the observed and expected prevalences in all subsamples in a prevalence curve.

Data preparation was done with SAS (Version 9.2). The simulation was calculated using R (version 2.12.0). Statistical analyses were made with Stata/MP (version 11.0) and figures were created using MS Excel 2003 SP 3.

The research expressed in this article was conducted according to the principles expressed in the Declaration of Helsinki. The researchers did not have to obtain informed consent, because the research was based on insurance claims data and the data set was analyzed anonymously (as regulated by German law in §75 SGB X). The study was approved by the Ethics Committee of the Medical Association of Hamburg including the waiver of consent (approval no. PV3057).

## Results

In total 121,389 persons were analyzed. Subsample 4 (which consists of persons with exactly 4 diseases from our list of 46) is the largest subsample in our data set with 18,075 patients (14.9%) and subsample 12 is the smallest subsample (with 1.2% of the total sample) and still includes 1,433 patients. The mean age of the total sample is 72.2 years. The lowest mean age can be found in subsample 1 (70.5 years) and the highest in subsample 12 (74.7 years). 56.4% of the total sample were male. Subsample 1 has the highest proportion of males (59.2%) and subsample 12 the lowest (52.2%).

Observed and expected prevalences and maximum deltas for 46 chronic conditions are shown in Table 2. The total sample includes all persons with at least 1 and not more than 12 chronic conditions. In this sample hypertension is the disease with the highest prevalence (63.1%) and hypotension (1.4%) has the lowest prevalence. There are six chronic conditions with a maximum delta ≥ +5.0% (hypertension, lipid metabolism disorders, chronic low back pain, atherosclerosis/PAOD, neuropathies and renal insufficiency) and also six with a maximum delta ≤ −5.0% (diabetes mellitus, thyroid dysfunction, severe vision reduction, cancers, prostatic hyperplasia and noninflammatory gynecological problems).

**Table 2:** Observed and expected prevalences and maximum deltas for 46 chronic conditions.

	total	subsample 1 disease		subsample 12 diseases		max. delta
	sample	observed	expected	observed	expected	(subsample)
***Hypertension***	***63.05***	***30.87***	***13.01***	***86.11***	***88.21***	***+23.69 (2)***
***Lipid metabolism disorders***	***39.63***	***5.61***	***8.30***	***72.02***	***74.51***	***+8.03 (4)***
***Chronic low back pain***	***36.25***	***7.59***	***7.55***	***79.13***	***71.55***	***+7.58 (12)***
***Diabetes mellitus***	***23.75***	***4.68***	***4.90***	***50.38***	***56.15***	***−5.77 (12)***
Joint arthrosis	23.15	3.01	4.82	57.64	55.18	+3.44 (10)
Chronic ischemic heart disease	20.78	2.88	4.33	49.69	51.31	−1.63 (12)
***Thyroid dysfunction***	***17.74***	***3.85***	***3.66***	***39.36***	***45.91***	***−6.56 (12)***
***Severe vision reduction***	***17.46***	***7.71***	***3.60***	***37.68***	***45.31***	***−7.63 (12)***
***Cancers***	***15.63***	***6.09***	***3.22***	***25.82***	***41.71***	***−15.89 (12)***
Cardiac arrhythmias	14.37	1.90	2.94	41.45	39.17	+2.28 (12)
Hyperuricemia/Gout	14.14	1.06	2.94	40.61	38.72	+2.61 (11)
***Prostatic hyperplasia***	***13.06***	***3.50***	***2.74***	***27.84***	***36.37***	***−8.52 (12)***
Lower limb varicosis	12.98	1.32	2.69	38.17	36.01	+2.16 (12)
Asthma/COPD	12.52	3.03	2.59	33.08	35.16	−2.65 (10)
***Atherosclerosis/PAOD***	***10.43***	***0.56***	***2.15***	***36.92***	***30.14***	***+6.77 (12)***
Depression	9.68	1.24	2.00	31.61	28.29	+3.33 (12)
Obesity	9.59	0.33	1.99	28.26	28.12	−2.40 (2)
Liver diseases	9.25	0.64	1.95	31.68	27.25	+4.43 (12)
Osteoporosis	8.62	1.23	1.82	24.35	25.49	−1.14 (12)
Chronic gastritis/GERD	8.50	1.09	1.74	26.24	25.40	−1.18 (3)
Cerebral ischemia/Chronic stroke	7.08	0.73	1.47	19.89	21.58	−1.70 (12)
Cardiac insufficiency	6.81	0.39	1.43	23.17	20.79	+4.30 (11)
***Noninflammatory gynaecologic problems***	***6.23***	***2.12***	***1.29***	***13.54***	***19.06***	***−5.52 (12)***
***Neuropathies***	***6.12***	***0.42***	***1.27***	***25.96***	***18.87***	***+7.08 (12)***
Chronic cholecystitis/Gallstones	5.81	0.36	1.21	17.24	18.04	−1.12 (3)
Allergies	5.71	0.59	1.18	17.79	17.76	−1.78 (10)
Insomnia	5.01	0.43	1.05	18.98	15.75	+3.23 (12)
Intestinal diverticulosis	4.75	0.34	1.02	17.24	14.97	+2.27 (12)
***Renal insufficiency***	***4.75***	***0.17***	***0.97***	***20.31***	***15.03***	***+5.27 (12)***
Hemorrhoids	4.63	0.45	0.96	17.31	14.66	+2.65 (12)
Somatoform disorders	4.52	0.38	0.96	15.98	14.30	+1.68 (12)
Cardiac valve disorders	4.34	0.30	0.89	14.72	13.71	+1.42 (10)
Urinary incontinence	3.85	0.22	0.78	11.86	12.26	+1.34 (12)
Dementias	3.64	1.20	0.75	10.33	11.63	−1.50 (12)
Severe hearing loss	3.60	0.72	0.74	13.61	11.47	+2.13 (12)
Dizziness	3.21	0.27	0.67	12.56	10.44	+2.12 (12)
Rheumatoid arthritis/Chronic polyarthritis	2.98	0.53	0.62	9.35	9.66	−1.18 (11)
Urinary tract calculi	2.77	0.12	0.57	9.84	8.99	+0.85 (12)
Anemias	2.67	0.15	0.55	10.33	8.64	+1.68 (12)
Migraine/chronic headache	2.52	0.25	0.52	7.47	8.15	−0.97 (10)
Psoriasis	2.21	0.58	0.45	5.72	7.19	−1.60 (11)
Sexual dysfunction	1.93	0.09	0.41	6.63	6.32	−0.68 (8)
Anxiety	1.91	0.20	0.39	8.44	6.24	+2.21 (12)
Tobacco abuse	1.49	0.09	0.31	4.61	4.89	−0.51 (8)
Parkinson's disease	1.48	0.54	0.30	4.75	4.87	−0.97 (10)
Hypotension	1.44	0.18	0.30	4.33	4.74	−0.84 (9)

Chronic conditions with a maximum delta > 5 or <−5 in bold and italic letters

**Figure 2:**
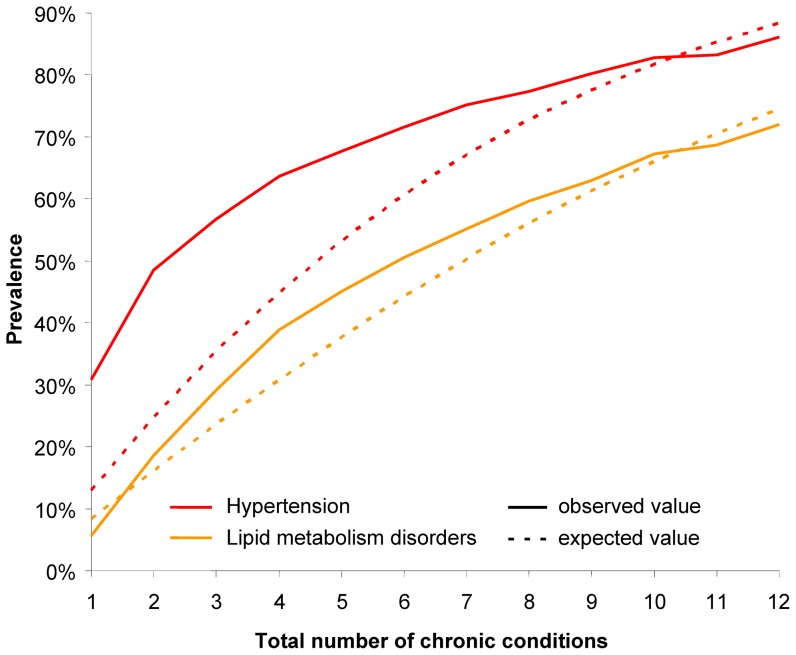
Observed and expected prevalences for hypertension and lipid metabolism disorders.

**Figure 3:**
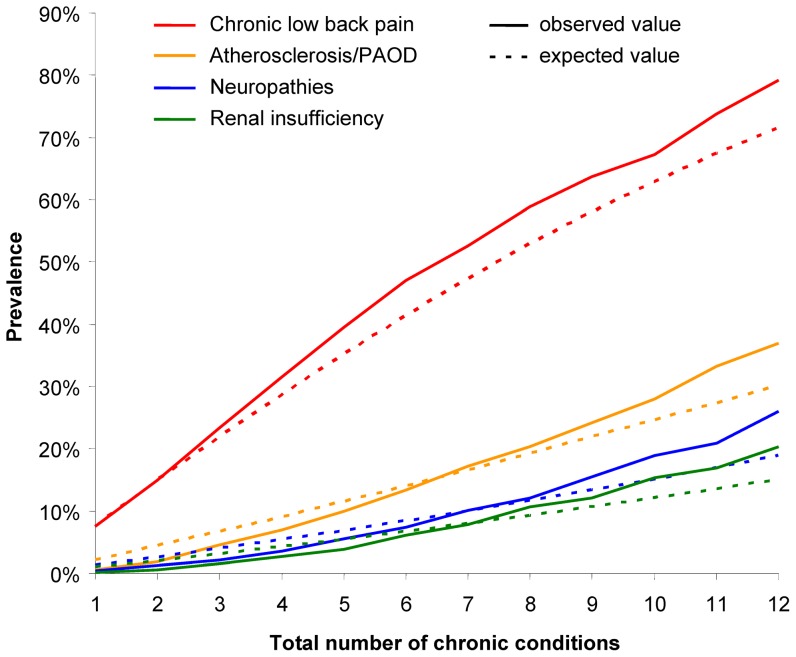
Observed and expected prevalences for chronic low back pain, atherosclerosis/PAOD, neuropathies and renal insufficiency.

Curves of observed and expected prevalences for chronic conditions with a maximum delta > +5% can be seen in Figure 2 and 3. Hypertension and lipid metabolism disorders have their maximum delta in the first six subsamples. The other four diseases have a higher than expected prevalence in subsamples 7 to 12 and (with the exception of chronic low back pain) are lower than expected in subsamples 1 to 6.

**Figure 4:**
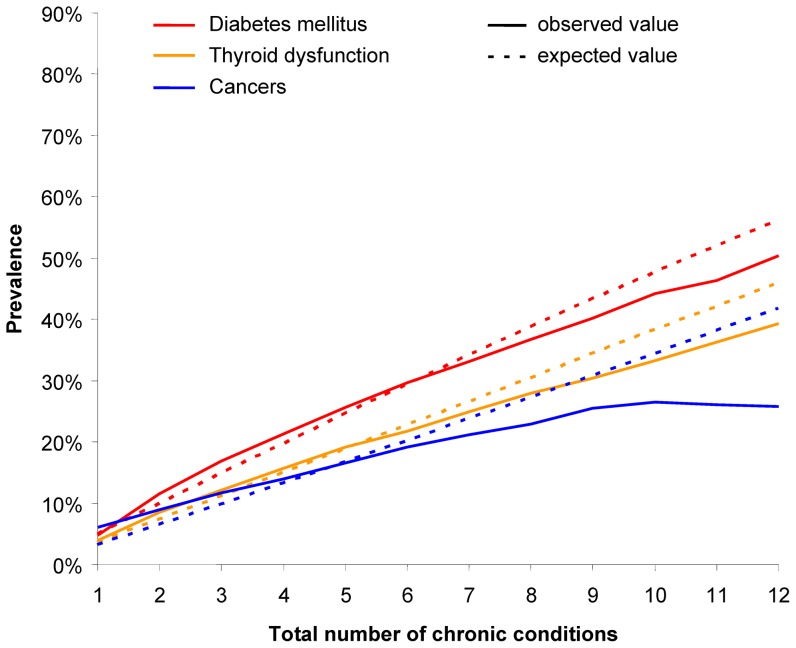
Observed and expected prevalences for diabetes mellitus, thyroid dysfunction and cancers.

**Figure 5:**
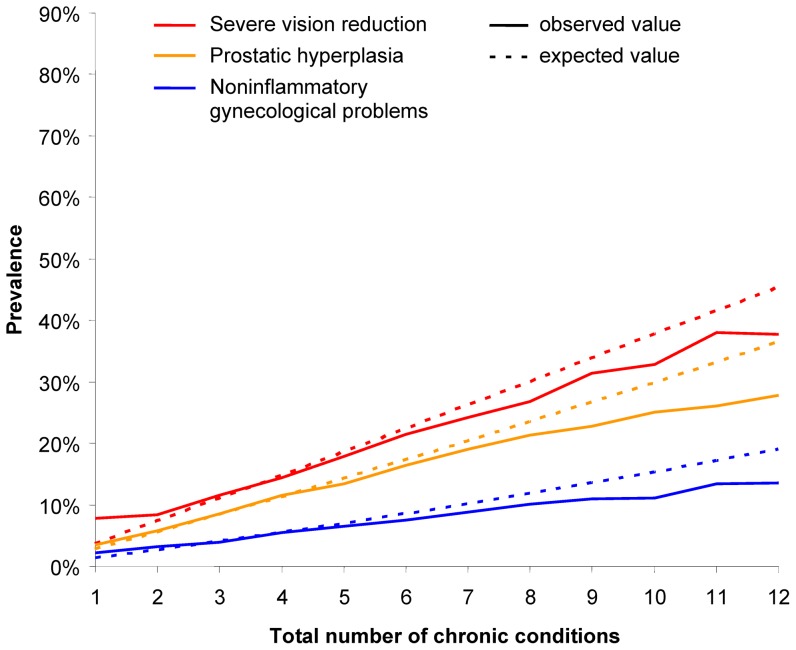
Observed and expected prevalences for severe vision reduction, prostatic hyperplasia and noninflammatory gynecological problems.

Curves of for chronic conditions with a maximum delta < −5% are shown in Figure 4 and 5. All six chronic conditions in these figure have a higher (or equal) than expected prevalence in the first four or five subsamples and a lower than expected prevalence in the other subsamples.

## Discussion

Only twelve of 46 chronic conditions show a frequency curve that differs in a clinically relevant extent from the curve that would be expected by chance. This does not necessarily mean that the occurrence rates of the other conditions are statistically independent from the number of chronic conditions as we did not compare incidence rates and time-to-event rates in a monomorbid population with rates of multimorbid patients. Instead, this finding can be interpreted in a way that these conditions do not exceed (or fall below) the average dependency between all chronic conditions. We also have to keep in mind that 19 of the 46 diseases have a total prevalence below 5% so that they are unlikely to reach the criterion of clinical relevance in the subsamples. The fact that renal insufficiency belongs to this group and nevertheless exceeds the relevance criterion shows the strong dependency of this condition on the number of comorbidities.

If we keep these limitations in mind we can classify the 28 remaining conditions (including renal insufficiency) in four types of growth: (1) *increased* prevalence in persons with a *low* number of comorbidities (i.e. in patients with less or equal than six chronic conditions), (2) *increased* prevalence in persons with a *high* number of comorbidities (i.e. in patients with more than six diseases), (3) *decreased* prevalence in persons with a *high* number of comorbidities, and (4) average course of prevalences.

Type (1) consists of hypertension and lipid metabolism disorders, which both are much more frequent than expected among persons with a relatively lower number of chronic conditions. These results do not surprise as both are known to be risk factors for a large number of chronic conditions like cardiovascular disease [13], [14], stroke [15], [16], and renal disorders [17], [18].

Type (2) includes atherosclerosis, neuropathies, renal insufficiency and chronic low back pain, which are all more frequent among persons with many diseases. This might be explained by the fact that most of these conditions are closely related to other chronic conditions, e.g. atherosclerosis can result from hypertension and hyperlipidemia [14]; and neuropathies and renal insufficiency are frequent complications of diabetes mellitus [19], [20].

Type (3) encompasses diabetes mellitus, cancers, thyroid dysfunction, severe vision reduction, noninflammtory gynecological problems and prostatic hyperplasia. All of these chronic conditions are less frequent than expected in persons with many comorbidities. This may result from three possibilities. First, they can be a precursor or early stage of other diseases. This especially applies to diabetes mellitus, which is known to be related to a large number of complications [21]. Second, a condition may result in very high mortality rates, so that the many patients die before they gain additional comorbidities. This may particularly be the case with cancer [22]. Third, it may be that the diseases are in fact less frequently (than average) related to other diseases.

Type (4) consists of 16 conditions that are related to an average extent to the other conditions. This is in many cases surprising as diseases like depression, chronic ischemic heart disease, stroke, gout and others have been shown to be dependent on a multitude of other chronic conditions [15], [23], [24]. This finding can be interpreted as an indicator for a high average interrelation with other diseases affecting most highly prevalent chronic conditions.

To our knowledge until now there have been no studies investigating the prevalence increase of chronic condition with a growing extent of multimorbidity, but there has been a previous study of our research team [4] using risk ratios for multimorbidity. This study identified that renal insufficiency, atherosclerosis and neuropathies were among the ten conditions with the highest risk ratio for multimorbidity. These results could now be confirmed. The chronic conditions obesity, liver diseases, chronic cholecystitis/gallstones and hyperuricemia/gout, which were also among this top ten list, only showed an average diathesis for multimorbidity in our present study. Hypertension, cancers and severe vision reduction were among the four conditions with the lowest risk for multimorbidity in our previous study. We now also found that these diseases occur more often in persons with less chronic conditions.

### Strengths and weaknesses

This study is the first to develop a stochastic model for comparing expected and observed prevalence rates in a multimorbid sample. The model fitted well as 16 of 28 diseases performed as expected. Because of the large sample size we refrained from testing for statistical significance. Instead we used a criterion for clinical relevance. In doing so we lost 18 of 46 diseases with a prevalence below 5%. These diseases were used for the stochastic model (and therefore for the simulation of the “expected” data set), but they could not be examined for comparison of expected and observed prevalences.

We were able to show that our model is less biased from the prevalence of the diseases than risk ratios for multimorbidity. Our comparisons can also discriminate between conditions that have a higher, a lower or an average association with the number of comorbidities of a patient. A problem of our approach lies in the fact that we cannot decide whether a disease is (absolutely) independent from other conditions.

Another limitation of our model lies in the fact that only bivariate comparisons were conducted. There are noticeable differences in age and gender between the subsamples and therefore these variables could confound our results. Conditions that are more frequent among women or very old patients might seem to have a higher association with the number of chronic conditions than they in fact have. As we have a cross-sectional study design our results could also be affected by selective survival, so that conditions with high morbidity rates could seem to be to a lesser extent associated with multimorbidity than they are in reality.

A strength of our approach relates to the selection of diseases. We included all highly prevalent chronic conditions (≥ 1% in the age group 65+) into our diagnosis groups and used them for our stochastic model. Our analyses are therefore based on a comprehensive picture of chronic diseases in individual patients.

Consideration must also be given to the data quality. Various studies have shown that there are differences in the distribution of age and gender [25] and in the prevalence of diseases [26] between the German health insurance companies. For this reason we compared the morbidity data of the Gmünder ErsatzKasse with data from a prospective cohort study of 3,189 patients in Germany [27]. This study has been published elsewhere. In short, we found that there may be an underreporting of diagnoses in the claims data from the Gmünder ErsatzKasse, but there was an acceptable correspondence of the relative prevalence and the rank order of the individual diseases between claims data and data from the cohort study. Because of the differences between data sources, studies relying on a single data source generally have to be interpreted with caution [28].

Although accidental and transitory diagnoses were excluded, in some cases diagnoses may be imprecise, ambiguous or incomplete because they were not clinically verified by trained professionals. This is a general problem in insurance claims data, but in our view, the benefits of claims data outweigh their disadvantages: We are provided with a large unselected population, representing real-world conditions and including persons living in protected institutions/nursing homes as well as frail individuals and the oldest olds, all frequently not included in survey and field studies. In choosing insurance claims data, we also avoided selection bias concerning service providers and as a matter of course there is no recall bias concerning diagnosis data.

### Conclusions

The growth of multimorbidity goes along with a rapid growth of prevalences in all chronic conditions. While this finding may be itself of importance for daily care of multimorbid patients it is – for the largest part – merely a stochastic effect. If we account for this effect we find that multimorbidity still seems to influence the occurrence rates of many chronic conditions, but in two directions: some conditions had a higher than expected prevalence and others had a lower than expected prevalence in patients with many comorbidities.

Our results also have methodological implications: We were able to show that the distribution of prevalences is complex and far from normality. If we use a naive approach for analyzing multimorbidity (e.g. by simply dividing the population in subsamples based on the number of chronic conditions without accounting for the distribution of diseases) these analyses might be affected by bias, because the prevalence of all chronic conditions necessarily increases with a growing extent of multimorbidity. For example, if disease burden is measured by the number of diseases of the individual patients and rare diseases in the study are more likely to produce a certain outcome, the effect of the disease count on this outcome can be confounded by the effect of the individual diseases. We should therefore always examine and discuss the close stochastic interrelations between the chronic conditions we include in our analyses.
